# The Effect of the Melt Viscosity and Impregnation of a Film on the Mechanical Properties of Thermoplastic Composites

**DOI:** 10.3390/ma9060448

**Published:** 2016-06-03

**Authors:** Jong Won Kim, Joon Seok Lee

**Affiliations:** Department of Textile Engineering and Technology, Yeungnam University, Gyeongsan 712-749, Korea; kjwfiber@ynu.ac.kr

**Keywords:** thermoplastic composite, impregnation, melt viscosity, pressure rising time, mechanical properties

## Abstract

Generally, to produce film-type thermoplastic composites with good mechanical properties, high-performance reinforcement films are used. In this case, films used as a matrix are difficult to impregnate into tow due to their high melt viscosity and high molecular weight. To solve the problem, in this paper, three polypropylene (PP) films with different melt viscosities were used separately to produce film-type thermoplastic composites. A film with a low melt viscosity was stacked so that tow was impregnated first and a film with a higher melt viscosity was then stacked to produce the composite. Four different composites were produced by regulating the pressure rising time. The thickness, density, fiber volume fraction (*V*_f_), and void content (*V*_c_) were analyzed to identify the physical properties and compare them in terms of film stacking types. The thermal properties were identified by using differential scanning calorimetry (DSC) and dynamical mechanical thermal analysis (DMTA). The tensile property, flexural property, interlaminar shear strength (ILSS), and scanning electron microscopy (SEM) were performed to identify the mechanical properties. For the films with low molecular weight, impregnation could be completed fast but showed low strength. Additionally, the films with high molecular weight completed impregnation slowly but showed high strength. Therefore, appropriate films should be used considering the forming process time and their mechanical properties to produce film-type composites.

## 1. Introduction

The application of carbon fiber reinforced plastic (CFRP) composites has been increasing with improved mechanical properties resulting from improved fibers and matrix materials, allowing them to displace conventional materials, such as steel, aluminum, and titanium alloys, for primary structures [1,2,3]. Fiber-reinforced thermoplastic (FRTP) composites have been applied widely to many industries owing to their advantages, such as fracture toughness, damage tolerance, ease of shape-forming before consolidation, significantly faster manufacturing, longer shelf life of raw materials, and the ability to be reshaped and reused [4]. On the other hand, the largest problem of thermoplastic composites is that the resin can barely penetrate the fiber bundles because of its high viscosity (500–5000 Pa·s) at melting temperatures when compared with that of thermosetting resins (commonly < 100 Pa·s) [5]. Many manufacturing processes for thermoplastic composites have been developed to solve the problem, and the use of a prepreg is the most common way to manufacture them [6,7,8,9]. 

A prepreg is where the reinforcing fibers or fabrics are pre-impregnated with resin. Thermoplastic prepregs undergo additional forming processes where needed; more than two prepregs are laminated and go through compression molding to produce the composite [10,11,12,13]. The property of prepregs could have a significant effect on the properties of the composite. The methods to produce a prepreg include film prepregging [14], hot melt prepregging [15,16], solution dip prepregging [17], powder prepregging [18,19], commingled prepregging [20], co-weaving prepregging [21], and *in-situ* polymerization [22,23]. 

For film prepregging, the pressure-temperature history of the polymer have the greatest effect on the impregnation (void content). For melting impregnation, however, this is impractical for certain polymers because of their limited tolerance to the temperatures necessary for viscosity reduction. Thermal degradation determined by a molecular weight reduction could be initiated within a few degrees of the melt temperature, making the viscosity reduction difficult or impractical [24]. In addition, there are mechanical limitations when using high pressures to impregnate a fiber. Generally, the mechanical properties of a composite deteriorate when a resin with low melt viscosity is used to improve impregnation owing to its low molecular weight. If a resin with a high degree of crystallization and a high molecular weight is used to improve the mechanical properties of a composite, impregnation decreases and reinforcement goes out of alignment owing to its high melt viscosity. In the case of using fabric-type reinforcement, the condition becomes worse. 

Common film-type prepregs are manufactured continuously through a roller or belt, and a considerable amount of research has been processed to reduce manufacturing time without any decrease in properties so as to lower the price. Melting the film without thermal degradation and impregnation of the reinforcing structure should be considered throughout the film-type prepreg manufacturing process. For films, the onset of melting, the rheological behavior at melt processing temperatures, and the time to drive impregnation should be considered [25,26,27].

In this paper, three polypropylene (PP) films with different melt viscosities were used separately to produce a composite. For the resin having low flowability with high melt viscosity, to reduce the time of driving impregnation and to improve the impregnation of the fiber, a film with a low melt viscosity was stacked so that tow was impregnated first; afterward, a film with a higher melt viscosity was stacked to produce the composite. The mechanical properties were then examined.

## 2. Materials and Methods

### 2.1. Materials

Woven fabric (SNC-1242R, plain, density (count/25 mm) = 6.4, weight = 420 g/m^2^, Seanal Tech-tex Co., Gumi, Korea) using carbon fiber (Toray T-300, 12K, Tokyo, Japan) was used as the reinforcing material. As the matrix, three PP films (SFI-151, SFI-740P, SFI-130A, Lotte Chemical Co., Seoul, Korea) with different melting flow indices (MFI) were used. Table 1 shows characterizations of the films.

### 2.2. Preparation of the Carbon Fiber-Reinforced Thermoplastic Composites

**Figure 1:**
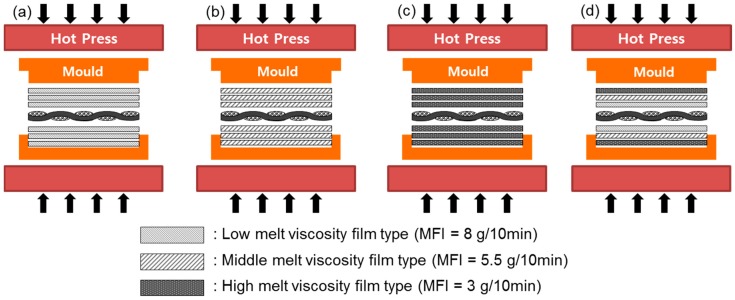
Schematic diagram of hot press processing: (**a**) low viscosity film type; (**b**) middle viscosity film type; (**c**) high viscosity film type and (**d**) combination (low-middle-high viscosity) film type.

The three PP films were stacked both above and under the carbon fiber fabric to produce the composite in the 4 stacking types, as shown in Figure 1. Only one type of film was used in the low melt viscosity type (LV-type), middle melt viscosity type (MV-type), and high melt viscosity type (HV-type); all three types of films were stacked for fiber impregnation in the order of the melt viscosity in the combination film type (CV-type). Those carbon fiber fabrics were laminated in 8 layers and inserted into a mold (295 × 295 mm^2^) for compression molding to produce the composite. After the mold was pre-heated in a hot press at 230 °C for 5 min, the pressure rising time was regulated by varying the time to reach 25 MPa from 5 to 25 min. When the time was shorter than 5 min, the reinforcement went out of alignment as a high pressure was suddenly applied so that the fiber could not impregnate the fabric, and voids could barely exit the inter-layer due to squeezing. The mold was then cooled to room temperature. The pressure was then removed and the composites were detached from the mold.

### 2.3. Measurement

The specimen thickness (mm) was averaged by measuring 4 edges and the center of each specimen using a thickness gauge. The fiber volume fraction (*V*_f_) of the specimen was calculated using a burn-off test according to ASTM D 2584. The specimen was inserted into a furnace in an inert environment for 10 h at 500 °C and dried in a desiccator. The *V*_f_ was then calculated based on the specimen weight ratio before and after the burn-off test. The actual density was calculated using the Archimedes principle according to ASTM D 792 by measuring the differences between the weight of a specimen in air and in water. The void volume content (*V*_c_) was calculated using Equation (1) based on the ASTM D 2734 as follows:

(1) $$V_{c}=100-\rho_{\text{sample}}\left(\frac{{\%\text{m}}_{\text{matrix}}}{\rho_{\text{matrix}}}+\frac{{\%\text{m}}_{\text{fiber}}}{\rho_{\text{fiber}}}\right),$$

where *V*_c_ is the void volume content (%), ρ is the primary density, and %m is the mass of each constituent.

The differential scanning calorimetry (DSC, Q-200, TA Instruments, Newcastle, DW, USA) patterns of specimens were monitored between 30 and 200 °C at a heating rate of 10 °C/min in a nitrogen atmosphere. The melting enthalpy was measured, and the degree of crystallinity (*X*_c_) was calculated using the following equation:

(2) $$X_{c\;}=\;\frac{\Delta H_{m}}{\Delta H^{{}^{\circ}}{}_{m}}\;\times \;\frac{100}{w}\;$$

where ΔH_m_ is the melting enthalpy calculated from the curve of melting endotherm of the sample, ΔH°_m_ (=209 J/g) is the enthalpy of the fusion of 100% crystalline PP, and w is the weight fraction of PP in the composite [28,29].

Dynamic mechanical thermal analysis (DMTA, Q-800, TA Instruments, Newcastle, DE, USA) was used to measure the viscoelastic properties of specimens. The single cantilever method was used for the specimens. The measurements were performed at a frequency of 1 Hz and amplitude of 1.5 µm. The temperature range was from 30 to 170 °C. The storage modulus (E’) and loss factor (tan δ) of the specimens were measured as a function of temperature. The tensile strength, flexural strength, and interlaminar shear strength (ILSS) were measured using a tensile tester (OTT-05, Oriental Co., Seoul, Korea) to determine the mechanical properties of the specimens. The tensile test was carried out according to ASTM D 3039 at a crosshead speed of 2 mm/min. A three-point-bending test was carried out based on the ASTM D 790 to measure the flexural strength with a span-to-depth ratio of 16 at a crosshead speed of 1.3 mm/min. The ILSS was also measured using a three-point-bending test based on the ASTM D 2344 with a span-to-depth ratio of 4 at a crosshead speed of 1 mm/min. The impregnation inside the tow was identified by scanning electron microscopy (SEM, S-4100, Hitachi, Tokyo, Japan).

## 3. Results and Discussion

### 3.1. Thickness and Fiber Volume Fraction

**Figure 2:**
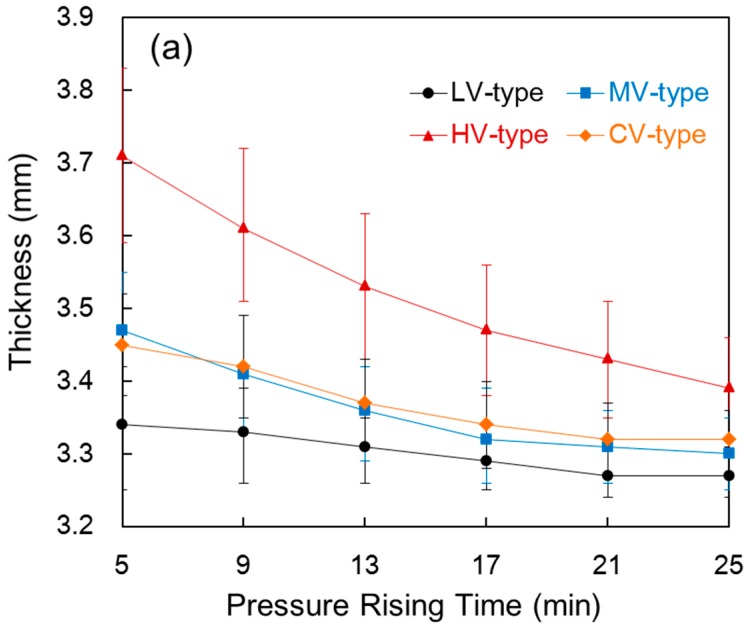

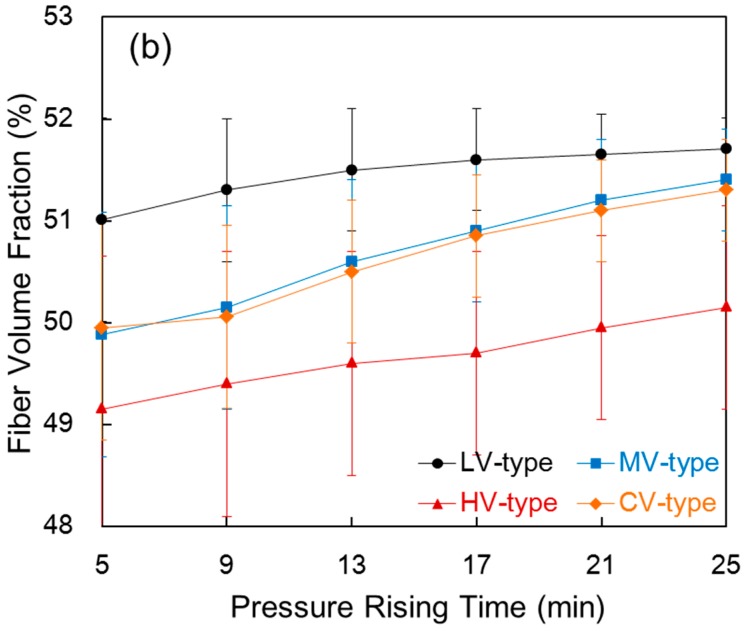
Thickness (**a**) and fiber volume fraction (**b**) of the composite compared with the pressure rising time in each film stacking type.

Figure 2 shows the thickness and fiber volume fraction of the composite compared with the pressure rising time in each film stacking type. In Figure 2a, the thickness of the composite decreased as pressure rising time increased. For the LV-type, thickness started to decrease slightly as the pressure rising time increased. This happened because tow impregnation and reinforcement of the composite were almost complete during the pre-heating process such that no resin came out. For the HV-type, the thickness of the composite is believed to continuously decrease because the unimpregnated fiber from the pre-heating process continued to undergo impregnation during the pressure rising process owing to its high melt viscosity. At a time of 25 min, the thickness of the HV-type was much higher than the LV-type, which is believed to happen since impregnation was not complete for the HV-type. The CV-type and MV-type showed a similar tendency. On the other hand, by comparing the thickness with the pressure rising time, at a shorter time of 5 min, the impregnation of tow was easier for a low melt viscosity film in the CV-type than the middle melt viscosity film in the MV-type, such that the composite was slightly thinner. At a longer time of 25 min, impregnation by the high melt viscosity film in the outer layer of the tow was much more difficult in the CV-type than that by the middle melt viscosity film in the MV-type because fiber impregnation was almost complete, such that the composite was slightly thicker.

In Figure 2b, the fiber volume fraction increased as pressure rising time increased. This happened because the resin, which is the matrix, was squeezed out from the composite due to the high pressure, since the fiber volume fraction is the only value that represents the volume of the reinforcement and matrix within the composite, regardless of the thickness of the composite. In the LV-type, the decrease in thickness and increase in fiber volume fractions were similar because almost no resin was squeezed out, as impregnation into the composite was almost complete. Therefore, the decrease in thickness was caused by the amount of squeezed out resin. On the other hand, the fiber volume fraction slightly increased when the thickness decreased, largely in the case of the HV-type. The resin appeared to be squeezed out from the mold, and impregnation into a large area of the unimpregnated tow within the composite occurred as the pressure rising time increased. An increase in fiber volume fraction in the MV-type and CV-type was larger than in the LV-type and HV-type. This happened because the amount of squeezed out resin was larger than the impregnation of unimpregnated fiber as the pressure rising time increased.

### 3.2. Density and Void Content

**Figure 3:**
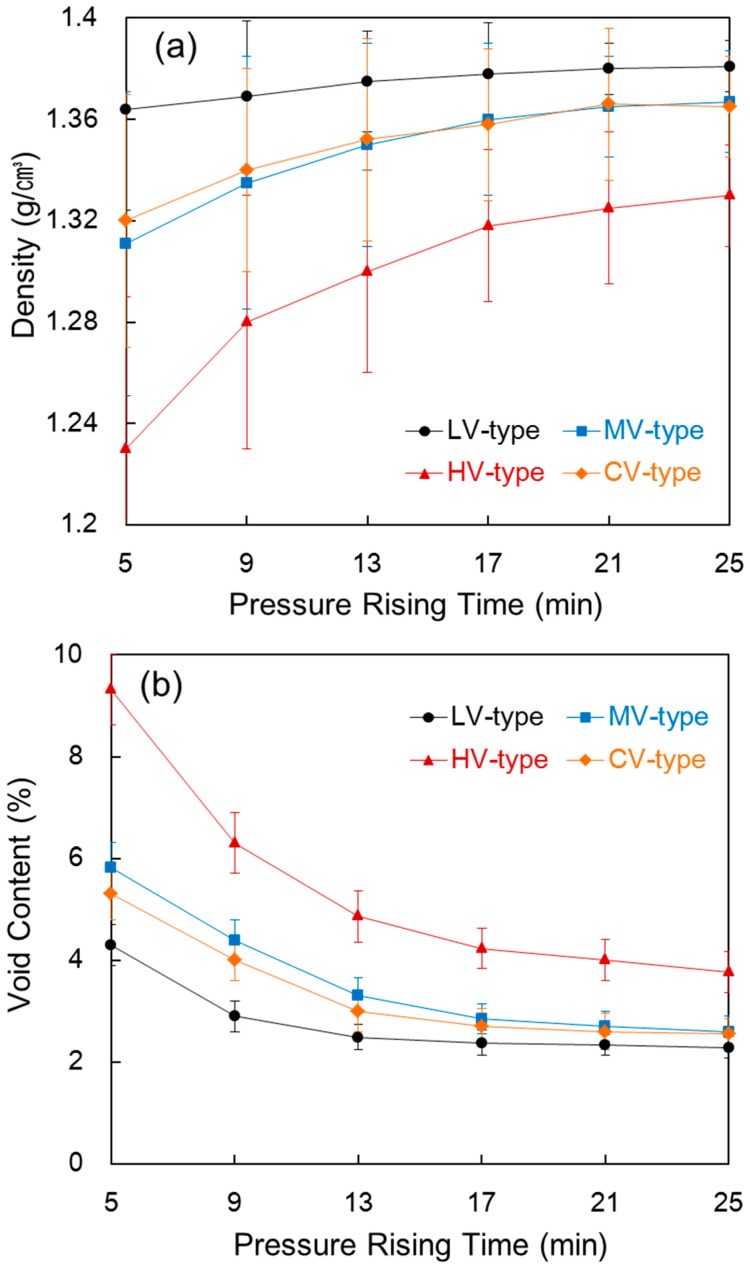
Density (**a**) and void content (**b**) of the composite compared with the pressure rising time in each film stacking type.

Figure 3 shows the density and void content of the composite compared with the pressure rising time in each film stacking type. As shown in Figure 3a, the density of the composite generally increased as pressure rising time increased. In the LV-type, the reason why density showed little change, even with a longer pressure time, is that the tow had already been sufficiently impregnated during the pre-heating process. In the HV-type, density rose as the pressure rising time increased because of the squeezed out resin from the decreased thickness and impregnation into the unimpregnated tow within the composite. In the MV-type and CV-type, density increased slightly as pressure rising time increased. This is due to the impregnation of the unimpregnated area within the tow and a decrease in thickness resulting from squeezed out resin.

As shown in Figure 3b, the void content also decreased as pressure rising time increased. The void content is the sum of the voids and unimpregnated area within the composite. This happened because, according to Equation (1), more impregnation was carried out during the pressure rising process for sufficient time so that the density of the composite increased. In the LV-type, the void content was 4.31% at a pressure rising time of 5 min, which means that the impregnation progressed considerably during the pre-heating process, and the decrease in void content is small because of the small amount of squeezed out resin, even when a longer pressure rising time is applied. On the other hand, the void content was 9.32% at a shorter pressure rising time of 5 min in the HV-type, so the unimpregnated area was considered large, and the void content was 3.77%, even at a time of 25 min. In the case of the HV-type, the high-melt-viscosity resin has low flowability, which makes impregnation of the tow difficult at shorter pressure rising times and also brings inadequate impregnation, even at a longer time. Similarly, in the MV-type and CV-type, the void content decreased as pressure rising time increased because the remaining unimpregnated area decreases and voids come out together when the resin is squeezed out.

### 3.3. Differential Scanning Calorimetry (DSC)

**Figure 4:**
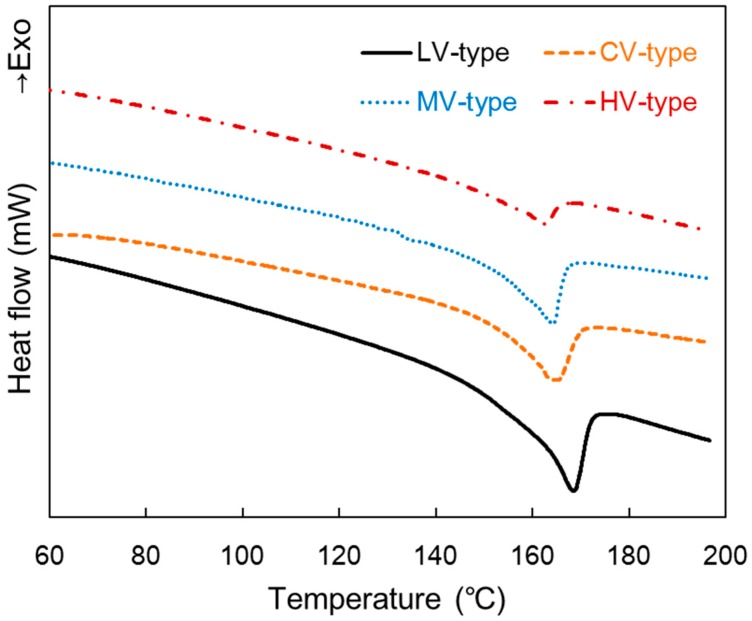
Differential scanning calorimetry (DSC) patterns of the composites produced at a pressure rising time of 25 min.

Figure 4 shows DSC patterns of the composites produced at a pressure rising time of 25 min. A film with higher melt viscosity showed a lower melting temperature peak, but the gap was small. The melting point of the CV-type was similar to the MV-type. *X*_c_ of the composite matrix, calculated according to Equation (2) based on the melting enthalpy of DSC, was 30.23% for the LV-type, 24.86% for the MV-type, 16.17% for the HV-type, and 26.92% for the CV-type. A film with higher molecular weight showed lower crystallinity of the matrix. Crystallinity in the CV-type is similar to the MV-type; however, the shape of the curve of the melting endotherm was slightly broad. This happened because three films with different molecular weights were combined [30]. 

### 3.4. Dynamical Mechanical Thermal Analysis (DMTA)

**Figure 5:**
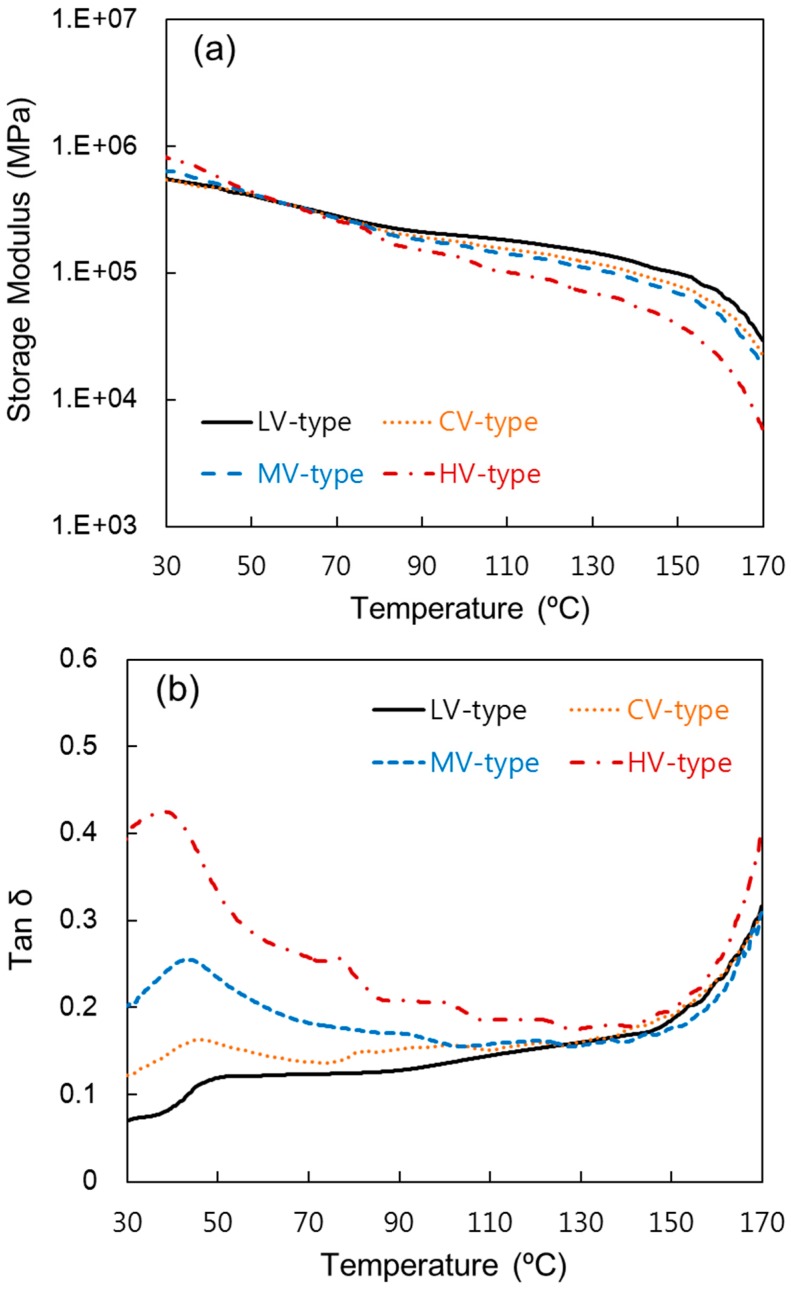
Dynamical mechanical thermal analysis (DMTA) of the composites produced at pressure rising time of 25 min. (**a**) Storage modulus; (**b**) loss factor (tan δ).

Figure 5 shows the viscoelastic properties of the composites measured by DMTA produced at a pressure rising time of 25 min. In Figure 5a, the storage moduli of all specimens decreased as temperature increased due to the softening of the matrix at a high temperature. The HV-type seems to be the stiffest at room temperature since its storage modulus is the highest. The storage modulus of the LV-type was the lowest at room temperature; however, the LV-type showed a slower downward trend as temperature rose. This shows that resin with low melt viscosity could achieve high adhesion. Therefore, heat-resistance was higher than other specimens [3,29]. 

As shown in Figure 5b, the loss factor (tan δ) provides information about the internal friction of the material and the adhesion of the interface. Broad peaks of the tan δ curve were shown scattered around 40 °C. The value of the peak of tan δ commonly shows the glass transition temperature (*T*g), and a higher *T*g was observed compared to the general PP. This might have been observed because it is fabric reinforcement composite—not a short fiber reinforcement composite. Films with low molecular weight showed a high tan δ peak temperature. This implies that films will become unstable over the temperature. In other words, the LV-type shows high heat resistance, as in the storage modulus curve [31,32].

### 3.5. Tensile Properties

**Figure 6:**
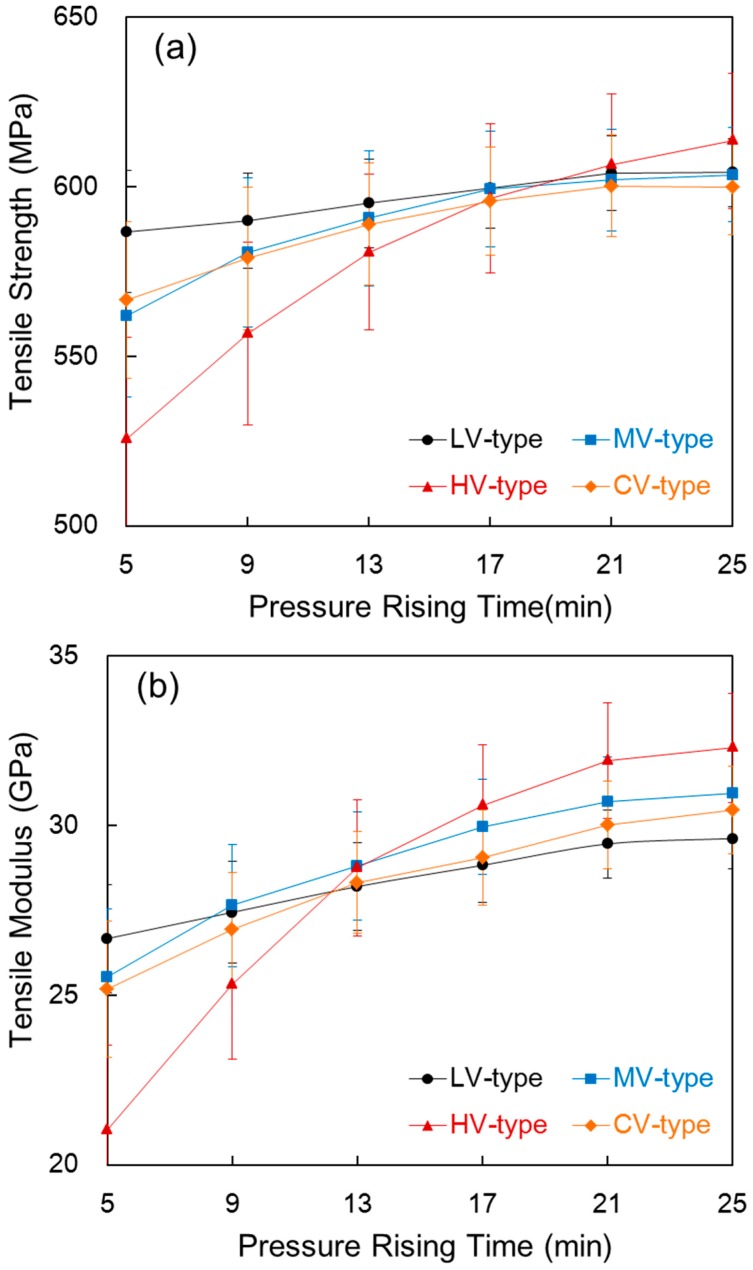
Tensile strength (**a**) and tensile modulus (**b**) of the composite compared with the pressure rising time in each film stacking type.

Figure 6 shows the tensile strength and tensile modulus of the composite compared with the pressure rising time in each film stacking type. In Figure 6a, the tensile strength generally rose as the pressure rising time increased. For the LV-type, since there was no big change in the fiber volume fraction of Figure 2b and in density of Figure 3a, which is because resin which melted during the pre-heating process due to its low melt viscosity promoted impregnation at a shorter pressure rising time, no more impregnation was processed, even with a longer pressure rising time, and surplus resin hardly came out, such that there was almost no change in thickness of the composite. In the HV-type, the tensile strength increased greatly as pressure rising time increased. The tensile strength increased considerably from 525.60 MPa to 613.56 MPa when the pressure rising time increased from 5 min to 25 min. As shown in Figure 2, this happened because the thickness of the composite decreased as the pressure rising time increased and impregnation then increased. The MV-type and CV-type showed a similar tendency towards increased tensile strength as the pressure rising time increased. This is due to the decrease in thickness caused by squeezed out resin and the slight increase in the fiber volume fraction as pressure rising time became longer. 

To compare the tensile strength of the composite with the film melt viscosity, at a pressure rising time of 5 min, the tensile strength of the HV-type with a higher melt viscosity film was 525.60 MPa, which was 10% lower than 586.82 MPa of the LV-type. This is apparently due to high thickness, in accordance with unimpregnation. On the other hand, at a pressure rising time of 25 min, the tensile strength of the HV-type was 613.56 MPa, which is higher than 604.28 MPa for the LV-type. In spite of a higher void content, a lower fiber volume fraction, and a higher thickness, the HV-type showed higher tensile strength than the LV-type. That is, the high melt viscosity film with a large molecular weight generally had a high tensile strength, such that impregnation of the composite was almost complete at a pressure rising time of 25 min, which indicates a contribution of the film strength. The difference in the tensile strength of the MV-type and CV-type was small, and their values were around the average of the LV-type and HV-type. Therefore, the tensile strength is affected by composite thickness resulting from impregnation throughout the pressure rising process.

As shown in Figure 6b, the tensile modulus in each melt viscosity of the film showed a similar tendency with tensile strength. On the other hand, the tensile modulus in the HV-type rose by 50% from 21.02 GPa to 32.29 GPa, unlike that in the LV-type, MV-type, and CV-type, when the pressure rising time increased from 5 min to 25 min. This is due to a decrease in strain at break caused by an increase in impregnation.

### 3.6. Flexural Properties

**Figure 7:**
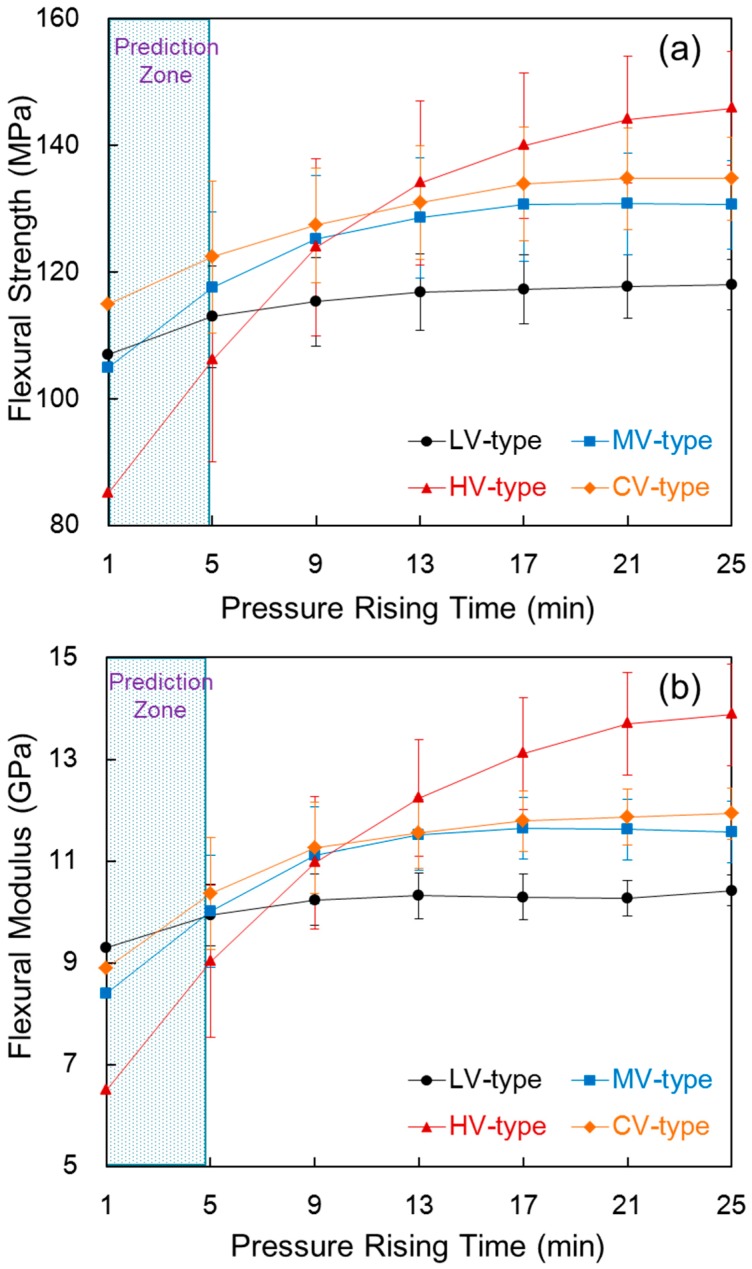
Flexural strength (**a**) and flexural modulus (**b**) of the composite compared with the pressure rising time in each film stacking type.

Figure 7 shows the flexural strength and flexural modulus of the composite compared with the pressure rising time in each film stacking type. As shown in Figure 7a, the flexural strength for all four composites increased as pressure rising time increased. In the LV-type, the flexural strength slightly increased as pressure rising time increased because the viscosity decreased enough to allow the low melt viscosity tow to be impregnated during the pre-heating process; thus, there were no large differences in impregnation, even with a longer pressure rising time. In the HV-type, the flexural strength increased greatly as the pressure rising time increased. When the pressure rising time increased from 5 min to 25 min, the flexural strength rose 39%, from 106.09 MPa to 145.84 MPa. This is a great increase compared to a decrease in composite thickness by 8.62%. This is due to the impregnation of the large unimpregnated area within the tow at a longer pressure rising time, and high-molecular-weight films have higher flexural properties than low-molecular-weight films. The flexural strengths of the MV-type and CV-type showed a similar tendency towards their tensile strengths. 

To compare the flexural strength of the composite with the film melt viscosity, at 5 min, the flexural strength of the HV-type was 106.09 MPa, which is 6.07% lower than 112.95 MPa of the LV-type. This happened because the HV-type is thicker and has a wide unimpregnated area. On the other hand, the difference in flexural strength was small compared to the 11.64% difference between the tensile strength of the HV-type and LV-type. This happened because, even in the case of using high melt viscosity film, impregnation occurs at a short pressure rising time and during the pre-heating process. In other words, flexural strength is strongly influenced by the binding force of the impregnation of the fiber and the matrix. At a longer pressure rising time of 25 min, the flexural strength of the HV-type was much higher at 145.84 MPa than 115.96 MPa for the LV-type. Although *V_c_* is high in the HV-type, it is due to an increase in the load to break, as impregnation was almost complete and thickness increased. That is, high mechanical properties of the film with a high molecular weight contributed to the flexural strength of the composite. In the CV-type and MV-type, the CV-type showed a higher flexural strength than the MV-type at all pressure rising times. Unlike in the MV-type, at a shorter pressure rising time of 5 min, impregnation first occurs in the case of low melt viscosity film, which means more fibers could be impregnated in the CV-type, resulting in high flexural strength. At a longer pressure rising time of 25 min, impregnation was almost complete for both types of films such that the outer layer of the tow in the CV-type appeared to be impregnated by high-melt-viscosity resin. As shown in the prediction zone under 5 min, impregnation occurred more in the CV-type during the pre-heating process than in the MV-type, and it occurs more easily during the pressure rising process as well. Thus, the LV-type showed low flexural strength when impregnation was completed fast, but the HV-type showed high flexural strength when impregnation was completed slowly.

As shown in Figure 7b, the flexural modulus showed a similar tendency with the flexural strength. Especially in the HV-type, the flexural modulus increased 53.76% from 9.02 GPa to 13.87 GPa when the pressure rising time increased from 5 min to 25 min. This happened because the composite became stiff, as the film with high molecular weight and tow completed impregnation when the pressure rising time increased. Therefore, the properties and impregnation of the film in the composite have a great effect on the flexural property.

### 3.7. Interlaminar Shear Stress (ILSS) 

**Figure 8:**
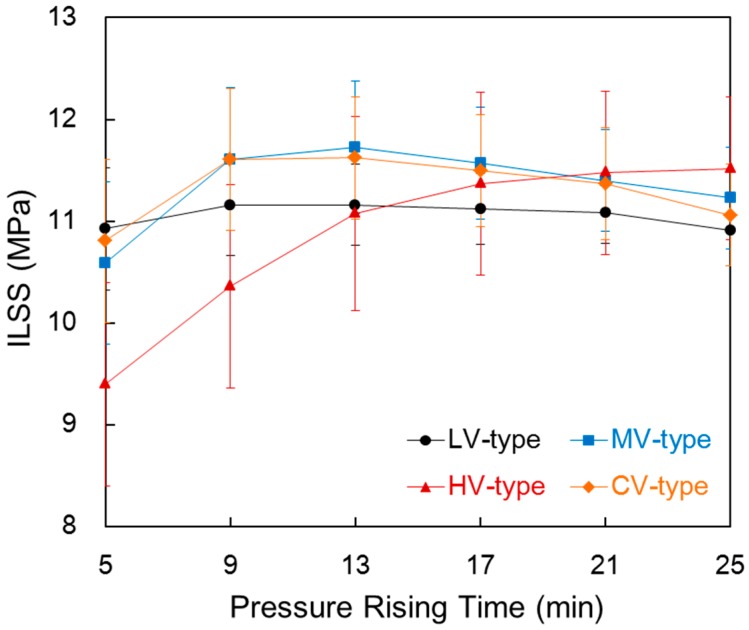
Interlaminar shear strength (ILSS) of the composite compared with the pressure rising time in each film stacking type.

Figure 8 shows the ILSS of the composite compared with the pressure rising time in each film stacking type. The ILSS of the four composites showed a different tendency as the pressure rising time increased. The ILSS in the LV-type showed almost no change in accordance with increases in pressure rising time. This is due to complete impregnation during the pre-heating process. The ILSS slightly decreased as the pressure rising time increased because of a small amount of resin that existed between the carbon fiber layers. ILSS in the HV-type increased continuously as pressure rising time increased. This is due to a decrease in thickness and *V*_c_ as impregnation increased. In the MV-type and CV-type, however, the ILSS increased until 13 min in the MV-type and 9 min in the CV-type, and then decreased thereafter. The ILSS implies the strength when the interface of the carbon fiber layers separate; therefore, it appears to increase at a shorter pressure rising time due to the decrease in thickness and void content, which reduces the unimpregnated area. With a longer pressure rising time, the layer becomes thinner as the resin between the carbon fiber layers is squeezed out after impregnation. The MV-type and CV-type in terms of the pressure rising time showed a similar tendency. ILSS was slightly lower in the CV-type than in the MV-type as pressure rising time increased. Here, impregnation was almost complete such that the resin within a carbon fiber layer had a higher stiffness in the CV-type than in the MV-type. However, the difference was small. Since the changes in thickness and fiber volume fraction were similar, the resin layers between the carbon fiber layers were similar.

### 3.8. Scanning Electron Microscopy (SEM)

**Figure 9:**
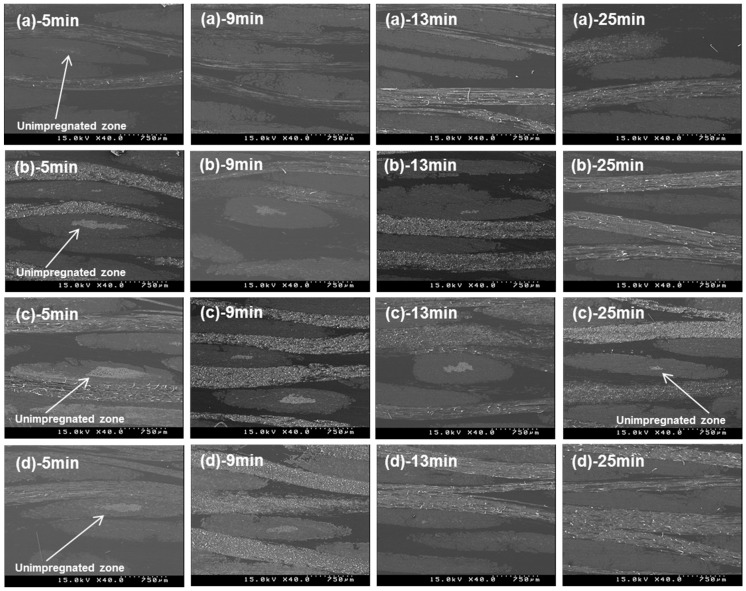
Cross-section SEM images in accordance with the pressure rising time in each film stacking type; (**a**) LV-type; (**b**) MV-type; (**c**) HV-type; (**d**) CV-type.

Figure 9 shows cross-section SEM images in accordance with the pressure rising time in each film stacking type. The LV-type showed complete impregnation at a shorter pressure rising time of 5 min. The HV-type showed a large unimpregnated area at 5 min and incomplete impregnation, even at a longer pressure rising time of 25 min. This shows that the melt viscosity of the film and pressure rising time affect impregnation. The MV-type and CV-type showed similar impregnation in terms of the pressure rising time; however, impregnation in the CV-type occurred slightly faster as the pressure rising time was increased from 5 min to 13 min. This is due to impregnation by the low melt viscosity film, which first occurred in the CV-type. 

## 4. Conclusions

To produce film-type thermoplastic composites, three PP films with different melt viscosities were used. A film with a low melt viscosity was stacked so that tow was impregnated first; then, a film with a higher melt viscosity was stacked to produce the composite. The mechanical properties of the composite were identified and the following conclusions were made.

As pressure rising time increased, the thickness of the four composites decreased due to squeezed out resin, increased fiber volume fraction, and increased density, and the *V*_c_ decreased as impregnation increased.

The tensile strength increased slightly in the LV-type, but greatly in the HV-type as pressure rising time increased. This increased similarly in the MV-type and in the CV-type. The tensile strength was affected by the composite thickness resulting from impregnation throughout the pressure rising process.

In the case of flexural strength, it increased slightly in the LV-type, but greatly in the HV-type as pressure rising time increased. The MV-type and CV-type showed a similar tendency, but the CV-type had higher values at each pressure rising time. LV-type showed low flexural strength when impregnation was completed fast, but HV-type showed high flexural strength when impregnation was completed slowly.

The ILSS mostly decreased, except in the case of the HV-type because the resin layer between the carbon fiber layers decreased as pressure rising time increased.

Appropriate films should be used considering the forming process time and their mechanical properties to produce film-type composites.

## Figures and Tables

**Table 1:** Characterizations of films from the manufacturer’s datasheets.

Properties	SFI-151	SFI-740P	SFI-130A
Melting flow index (g/10 min)	8	5.5	3
Molecular weight (g/mol)	250,000	270,000	300,000
Melting point (°C)	166.1	162.6	160.3
Density (g/cm^3^)	0.90	0.90	0.90
Tensile stress at yield (MPa)	21	26	32
Tensile strain at break (%)	>500	>500	>500
Flexural modulus (MPa)	670	980	1470
Thickness (µm)	50	50	50
